# Algoritmo de manejo hospitalario para la intoxicación aguda por Paraquat^®^ en población pediátrica, serie de casos

**DOI:** 10.7705/biomedica.7024

**Published:** 2024-03-31

**Authors:** Alejandro Padilla-Guzmán, Olga L. Prado, David Ballesteros, Viviana Rivera, Yessica Bravo, Luisa Murillo, Sandra Narváez, Jessica M. Forero

**Affiliations:** 1 Centro de Investigaciones Clínicas, Fundación Valle del Lili, Cali, Colombia Fundación Valle del Lili Fundación Valle del Lili Cali Cali; 2 Nefrología y Cuidado Intensivo Pediátrico, Hospital Universitario San José, Popayán, Colombia Hospital Universitario San José Hospital Universitario San José Popayán Popayán; 3 Servicio de Nefrología Pediátrica, Fundación Valle del Lili, Cali, Colombia Fundación Valle del Lili Fundación Valle del Lili Cali Cali; 4 Facultad de Ciencias de la Salud, Universidad Icesi, Cali, Colombia Universidad Icesi Universidad Icesi Cali Cali

**Keywords:** Paraquat^®^, intoxicación, niños, hemoperfusión, inmunosupresores, Paraquat^®^, poisoning, child, hemoperfusion, immunosuppressive agents

## Abstract

El dicloruro de 1,1’-dimetil-4,4’-bipiridilo (Paraquat^®^) es un compuesto químico de la familia de las piridinas, utilizado como herbicida no selectivo y desecante. Este compuesto puede causar intoxicación aguda por todas las vías de exposición. En el momento, no hay un antídoto conocido y los tratamientos disponibles, incluidos los pediátricos, se basan en contrarrestar su absorción y propiciar su remoción oportuna.

Se describe una serie de casos de 14 pacientes pediátricos, procedentes en su mayoría del departamento del Cauca, con intoxicación aguda por ingestión de paraquat. Los pacientes fueron remitidos y atendidos en un hospital de mediana a alta complejidad en el suroccidente colombiano, con un protocolo institucional para el manejo de la intoxicación aguda por el herbicida.

La intoxicación aguda con paraquat por vía oral se asocia con una alta tasa de mortalidad, aún con atención médica oportuna, pues fácilmente se alcanzan concentraciones sistémicas para ser fulminante. Basado en la literatura disponible, el Hospital Universitario San José ha propuesto un protocolo clínico -adecuado para la intoxicación aguda por paraquat en población pediátrica- que incluye manejo estándar temprano, tratamiento inmunosupresor y antioxidante, y técnicas para su remoción sistémica

## Abstract (en)

Paraquat®, or N,N‘-dimethyl-4,4‘-bipyridinium *dichloride*, is a bipyridyl compound used as a non-selective herbicide and desiccant that can cause acute poisoning through all routes of exposure. There is no known antidote, and the available treatments are based on avoiding its absorption and timely removing it, in adults and children.

We describe a case series of 14 pediatric patients from the department of Cauca, Colombia, with acute intoxication after oral intake of paraquat. Patients were referred to a medium-high complexity hospital in southwestern Colombia and treated according to an institutional protocol for acute paraquat poisoning. Acute paraquat poisoning after oral ingestion is associated with a high mortality rate, even with timely medical attention, as the compound has no known antidote and quickly reaches systemic concentrations for fulminant poisoning. Based on the available literature, our center has proposed a clinical protocol including early standard management, immunosuppressive and antioxidant treatments, and systemic removal techniques. This protocol suggests an adequate approach to acute paraquat poisoning in the pediatric population.

El Paraquat^®^ (dicloruro de 1,1’-dimetil-4,4’-bipiridilo o dimetilsulfato de 1,1’dimetil4,4’-bipiridilo) es un compuesto químico de la familia de las piridinas, utilizado como herbicida no selectivo y desecante ^1^^-^^3^. Se clasifica como un pesticida de uso restringido en diversos países para el control de malezas y pastos en áreas agrícolas y no agrícolas ^2^^,^^4^; se encuentra en estado líquido, a una concentración del 20 % ^5^. Este compuesto puede causar intoxicación aguda por cualquier vía de exposición ^2^^,^^4^ y es sumamente hidrofílico ^3^.

Se ha reportado un elevado número de intoxicaciones, tanto accidentales como intencionales ^2^^,^^4^, asociadas con una importante tasa de mortalidad aún con atención médica oportuna ^1^^,^^6^. En el momento, no hay un antídoto conocido y los tratamientos disponibles se basan en contrarrestar la absorción del compuesto y en propiciar su remoción oportuna ^3^^,^^7^^-^^10^. Otras alternativas terapéuticas, como el tratamiento inmunosupresor y antioxidante, están en debate y carecen de evidencia clínica contundente a su favor ^3^^,^^7^^,^^11^.

Se describe una serie de casos de intoxicación por paraquat en población pediátrica, tratados según un protocolo institucional. También, se hace una revisión de la intoxicación aguda por paraquat a pesar de la escasa literatura disponible, en particular, en población pediátrica.

## Descripción de los casos

El resumen de las variables sociodemográficas y clínicas de los casos se encuentra en el cuadro 1.

**Cuadro 1:** Variables sociodemográficas y clínicas de los casos pediátricos de intoxicación aguda por paraquat (N = 14)

Variables	
Edad (años) Sexo		Mediana: 16,00; RIC: 14,00-16,00
	Masculino	6
	Femenino	8
Procedencia		
	Argelia (Cauca)	2
	Morales (Cauca)	1
	Popayán (Cauca)	2
	Departamento del Putumayo	1
	El Tambo (Cauca)	2
	Toribío (Cauca)	6
Cantidad ingerida de paraquat (ml) (n=11)		Mediana: 15,00; RIC: 10,00-20,00
Tiempo de inicio de síntomas (horas) (n=3) Síntomas iniciales		Mediana: 5,00; RIC: 5,00-50,50
	Emesis	9
	Náuseas	2
	Dolor abdominal	5
	Parestesias	2
	Diarrea	1
	Sudoración	1
	Malestar general	2
	Lesiones ulcerativas en mucosa oral	1
	Disfagia	1
	Somnolencia	1
	Dolor torácico	1
	Sialorrea	2
	Síncope	1
	Mareo	1
	Odinofagia	1
	Fasciculaciones	1
Tiempo entre ingestión e ingreso (horas)		Mediana: 11,00; RIC: 7,50-22,00
Manejo inicial antes del ingres		
	Carbón activado	8
	Lavado gástrico	6
	Líquidos intravenosos	6
	Protección gástrica	4
	Esteroides	1
	Sin datos	1
	Sin manejo	1
Frecuencia cardiaca al ingreso (lpm)		Media: 90,43 ± 18,49
Frecuencia respiratoria al ingreso (rpm)		Media: 21,43 ± 5,11
Saturación de oxígeno al ingreso		Media: 96,86 ± 1,17
Hemoperfusión con filtro de carbón activado		12
Tiempo desde el ingreso e inicio de hemoperfusión con carbón activado (horas)		Media: 6,92 ± 3,78
Tipo de cartucho usado para hemoperfusión con carbón activado (n=12)		
	Adsorba 300C®	12
Número de sesiones para hemoperfusión con carbón activado (n=12)		
	1	8
	2	4
Duración de hemoperfusión con carbón activado (horas)		Mediana: 4; RIC: 4-6
Hemodiafiltración veno-venosa continua		13 (92,9)
Duración de la hemodiafiltración veno-venosa continua (horas)		Media: 73,9 ± 25,7
Tipo de cartucho usado para hemodiafiltración veno-venosa continua (n=13)		
	ST100-150®	12
	HF20^®^	1
Asistencia respiratoria mecánica		3
Soporte inotrópico		3
Soporte vasopresor		3
Hemodiálisis		4
Tiempo entre ingestión y muerte (horas) (n=3)		Mediana: 4,00; RIC: 4,00-4,50
Complicaciones mayores		
	Perforación o quemadura esofágica	4
	Bacteriemia	
	Lesión renal aguda	2
	Fibrosis pulmonar	3
	Neumonía aspirativa	2
	Neumonitis química	2
Duración de hospitalización en la unidad pediátrica de cuidados intensivos (días)		Mediana: 10; RIC: 5,5-14

lpm: latidos por minuto; rpm: respiraciones por minuto; RIC: rango intercuartílico

Se describen 14 casos de pacientes pediátricos con intoxicación aguda por paraquat, con edades entre 1 y 17 años, la mayoría procedentes de otros municipios del departamento del Cauca diferentes a su capital, Popayán. El 71 % recibió atención médica en las primeras 24 horas de la ingestión del paraquat y todos los casos presentaron algún tipo de sintomatología. La emesis y el dolor abdominal fueron los síntomas más frecuentes.

El tratamiento más común antes del ingreso a la institución incluyó lavado gástrico, administración de carbón activado, líquidos intravenosos y protección gástrica. Todos los pacientes recibieron el tratamiento farmacológico descrito en el protocolo, pero, además, 12 recibieron hemoperfusión con carbón activado y 13, hemodiafiltración veno-venosa continua. El sistema utilizado para las terapias fue Prismaflex®, versión 8.2. El filtro utilizado para todas las terapias de hemoperfusión fue Adsorba 300C® y, para las de hemodiafiltración, fue HF20 o ST100-150 según la superficie corporal del paciente. Después de administrar el tratamiento según el protocolo institucional, la mortalidad fue del 21 %. Los casos se describen con detalle a continuación.

### Caso 1

Se trata de un paciente de 16 años de sexo masculino, procedente de la zona rural de El Tambo (Cauca), sin antecedentes clínicos referidos, que ingresó al Hospital Universitario San José 72 horas después de ingerir “dos tragos” de paraquat (aproximadamente 20 ml), con náuseas, emesis, dolor abdominal y parestesias que se iniciaron a las cinco horas de la ingestión.

Inicialmente, fue tratado con líquidos endovenosos y protección gástrica en un centro médico de nivel I de atención. Al llegar al Hospital Universitario San José presentaba tensión arterial sistólica de 117 mm Hg y diastólica de 61 mm Hg, frecuencia cardiaca de 71 latidos por minuto, frecuencia respiratoria de 23 respiraciones por minuto y saturación de oxígeno del 96 %. El paciente fue trasladado a la unidad pediátrica de cuidados intensivos, en donde se le administró metilprednisolona, ciclofosfamida, colchicina, N-acetilcisteína, vitamina E, ácido ascórbico y vitamina A. No recibió hemoperfusión con carbón activado, pues el tiempo transcurrido después de la ingestión del tóxico ya era mayor de 24 horas, pero recibió hemodiafiltración veno-venosa continua por 48 horas. Durante su estancia en la unidad pediátrica de cuidados intensivos, se identificaron quemaduras en la vía digestiva, por lo cual el paciente requirió asistencia respiratoria mecánica, y soporte inotrópico y vasopresor.

El paciente falleció a los cuatro días después de la ingestión de paraquat.

### Caso 2

Se trata de un paciente de 14 años de sexo masculino, procedente de la zona rural de El Tambo (Cauca), sin antecedentes clínicos referidos, que ingresó a la institución a las 18 horas después de ingerir “dos sorbos” de paraquat, con síntomas que se iniciaron a las cinco horas de la ingestión, como emesis, diarrea, sudoración, malestar general y parestesias.

Inicialmente, fue tratado con líquidos endovenosos y esteroides en un centro médico de nivel I de atención. Al llegar al Hospital Universitario San José, presentaba tensión arterial sistólica de 136 mm Hg y diastólica de 94 mm Hg, frecuencia cardiaca de 131 latidos por minuto, frecuencia respiratoria de 22 respiraciones por minuto y saturación de oxígeno del 96 %.

El paciente fue trasladado a la unidad pediátrica de cuidados intensivos, en donde recibió metilprednisolona, ciclofosfamida, colchicina, N-acetilcisteína, vitamina E, ácido ascórbico y vitamina A. Transcurridas dos horas desde su ingreso, se inició hemoperfusión con carbón activado y, luego, hemodiafiltración veno-venosa continua por 72 horas. Durante su estancia en la unidad de cuidados intensivos, se identificó perforación esofágica, por lo que el paciente requirió asistencia respiratoria mecánica, y soporte inotrópico y vasopresor.

El paciente falleció cuatro días después de la ingestión de paraquat.

### Caso 3

Se trata de una paciente de 16 años de sexo femenino, procedente de la zona rural de Toribío (Cauca) sin antecedentes clínicos referidos, que ingresó a la institución a las 120 horas después de ingerir de 5 a 10 ml de paraquat, con lesiones ulcerativas en mucosa oral, disfagia y dolor torácico.

Inicialmente, fue tratada con líquidos endovenosos y protección gástrica en un centro médico de nivel I de atención. Al llegar al Hospital Universitario San José, presentaba tensión arterial sistólica de 120 mm Hg y diastólica de 70 mm Hg, frecuencia cardíaca de 77 latidos por minuto, frecuencia respiratoria de 16 respiraciones por minuto y saturación de oxígeno del 95 %.

La paciente fue trasladada a la unidad pediátrica de cuidados intensivos en donde se le administró metilprednisolona, ciclofosfamida, colchicina, N-acetilcisteína, vitamina E, ácido ascórbico y vitamina A. No recibió hemoperfusión con carbón activado, ni hemodiafiltración veno-venosa continua pues ya había pasado el periodo para ello y por la ausencia de desarrollo de lesión renal aguda. Durante su estancia en la unidad pediátrica de cuidados intensivos, no requirió asistencia respiratoria mecánica, ni soporte inotrópico o vasopresor.

La paciente desarrolló bacteriemia por *Enterobacter cloacae* que se trató con antibióticos y, finalmente, se recuperó y egresó del hospital.

### Caso 4

Se trata de una paciente de un año de sexo femenino, procedente del departamento del Putumayo, sin antecedentes clínicos conocidos, que ingresó a la institución a las 48 horas de ingerir una cantidad desconocida de paraquat (ropa y boca impregnadas), con emesis inmediata al hacerlo.

Inicialmente, fue tratada con líquidos endovenosos y protección gástrica en un centro médico de nivel I de atención y, luego en uno de nivel II, con metilprednisolona y N-acetilcisteína. Al llegar al Hospital Universitario San José, presentaba tensión arterial sistólica de 88 mm Hg y diastólica de 56 mm Hg, frecuencia cardíaca de 120 latidos por minuto, frecuencia respiratoria de 28 respiraciones por minuto y saturación de oxígeno del 98 %.

La paciente fue trasladada a la unidad pediátrica de cuidados intensivos, en donde recibió metilprednisolona, ciclofosfamida, colchicina, N-acetilcisteína, vitamina E, ácido ascórbico y vitamina A. Transcurridas seis horas desde su ingreso a la institución, se inició hemoperfusión con carbón activado y, luego, hemodiafiltración veno-venosa continua por 72 horas. Durante su estancia en la unidad pediátrica de cuidados intensivos, no requirió asistencia respiratoria mecánica, pero sí soporte inotrópico y vasopresor, debido a un episodio de actividad eléctrica sin pulso por desequilibrio electrolítico.

La paciente se recuperó y egresó del hospital.

### Caso 5

Se trata de una paciente de 17 años de sexo femenino, procedente de zona rural de Toribio (Cauca), con antecedentes de obesidad, que ingresó a la institución a las cinco horas de haber ingerido una cantidad desconocida de paraquat, con posterior somnolencia y sin otros síntomas.

Inicialmente, se le practicó lavado gástrico, con administración de carbón activado por sonda nasogástrica, infusión de líquidos endovenosos y protección gástrica, en un centro médico de nivel I de atención. Al llegar al Hospital Universitario San José, presentaba frecuencia cardíaca de 109 latidos por minuto, frecuencia respiratoria de 22 respiraciones por minuto, saturación de oxígeno del 98 %, tensión arterial sistólica de 133 mm Hg y diastólica de 64 mm Hg.

Se inició el tratamiento con metilprednisolona e ivermectina, y se trasladó a la unidad pediátrica de cuidados intensivos, donde recibió metilprednisolona, ciclofosfamida, colchicina, N-acetilcisteína, vitamina E, ácido ascórbico, vitamina A y propanolol. Diez horas después de su ingreso, se le practicó hemoperfusión con carbón activado y luego, hemodiafiltración veno-venosa continua por 72 horas. Durante su estancia en la unidad pediátrica de cuidados intensivos, no requirió asistencia respiratoria mecánica, ni soporte inotrópico o vasopresor, a pesar de las quemaduras esofágicas y una lesión renal aguda.

La paciente presentó mejoría clínica y egresó a los 20 días de hospitalización en buenas condiciones generales.

### Caso 6

Se trata de un paciente de 16 años de sexo masculino, procedente de Toribio (Cauca), que ingresó a la institución a las cuatro horas después de haber ingerido una cantidad desconocida de paraquat. Los familiares del menor lo encontraron inconsciente y con sialorrea, y lo llevaron a un centro médico de nivel I de atención, donde fue tratado con carbón activado y líquidos endovenosos.

Al llegar al Hospital Universitario San José, presentaba tensión arterial sistólica de 110 mm Hg y diastólica de 65 mm Hg, frecuencia cardíaca de 99 latidos por minuto, frecuencia respiratoria de 16 respiraciones por minuto y saturación de oxígeno del 96 %. Presentó un puntaje de 7/15 en la escala de Glasgow, sialorrea importante y fasciculaciones generalizadas, por lo que se le sometió a intubación orotraqueal. Se sospechó una intoxicación por organofosforados por el aparente síndrome tóxico nicotínico y se activó el protocolo para la intoxicación con paraquat. El paciente se trasladó a la unidad pediátrica de cuidados intensivos y se le administró ácido ascórbico, vitamina A, vitamina E, colchicina, albendazol, ivermectina, metilprednisolona, ciclofosfamida y propanolol. Se inició hemoperfusión con carbón activado a las seis horas del ingreso y, luego, hemodiafiltración veno-venosa continua por 72 horas. Se diagnosticó neumonía aspirativa (con probable fibrosis pulmonar), que fue tratada con ampicilina-sulbactam. El paciente no requirió soporte inotrópico o vasopresor y fue extubado al tercer día, pero posteriormente desarrolló fiebre, signos de falla orgánica multisistémica y lesión renal aguda, y falleció al quinto día del ingreso hospitalario.

### Caso 7

Se trata de un paciente de 13 años de sexo masculino, procedente de la zona rural de Toribio (Cauca), y sin antecedentes clínicos referidos, que ingresó a la institución cuatro horas después de haber ingerido 10 ml de paraquat, con síntomas de mareo, emesis y dolor abdominal.

Inicialmente, fue trasladado a un centro médico de nivel I de atención, donde fue tratado con carbón activado y fue remitido como urgencia vital. Al llegar al Hospital Universitario San José, presentaba regular estado general, tensión arterial sistólica de 121 mm Hg y diastólica de 85 mm Hg, frecuencia cardíaca de 83 latidos por minuto, frecuencia respiratoria de 21 respiraciones por minuto y saturación de oxígeno del 96 %; además, nistagmo horizontal, pupilas midriáticas de 0,8 mm, y se encontró orientado en las tres esferas, alerta, bradipsíquico y bradilálico. El menor refirió que una semana atrás estuvo expuesto a glifosol, un compuesto tóxico.

Se trasladó a la unidad pediátrica de cuidados intensivos, donde se inició tratamiento con ciclofosfamida, N-acetilcisteína, metilprednisolona, propanolol, ondansetrón, vitamina C, vitamina A, vitamina E, enoxaparina, ivermectina y albendazol. Se practicó hemoperfusión con carbón activado siete horas después del ingreso y, luego, hemodiafiltración veno-venosa continua durante 88 horas. No requirió asistencia respiratoria mecánica invasiva, ni soporte inotrópico o vasopresor. En la endoscopia de vías digestivas altas, se encontró gastritis antrocorporal erosiva y quemadura en el esófago distal. Se indicó tratamiento con ampicilina-sulbactam por riesgo de mediastinitis y nutrición por sonda nasoyeyunal con suplemento; luego, su tolerancia a la vía oral fue adecuada. Psiquiatría infantil indicó manejo para depresión y, dada su evolución clínica positiva, el paciente egresó del hospital después de 18 días.

### Caso 8

Se trata de un paciente de 16 años de sexo masculino, procedente de Suárez (Cauca), que ingresó a un centro médico de nivel I de atención a las tres horas de haber ingerido aproximadamente 10 ml de paraquat.

Le practicaron lavado gástrico, le administraron carbón activado y lo remitieron como urgencia vital al Hospital Universitario San José 10 horas después de la ingestión del tóxico. El paciente refirió odinofagia y dolor abdominal en el mesogastrio, sin otros síntomas. Presentaba tensión arterial sistólica de 114 mm Hg y diastólica de 79 mm Hg, frecuencia cardíaca de 70 latidos por minuto, frecuencia respiratoria de 20 respiraciones por minuto y saturación de oxígeno del 99 %.

Se inició tratamiento con metilprednisolona, ciclofosfamida, N-acetilcisteína, vitamina E, vitamina C, omeprazol y albendazol. Fue trasladado a la unidad pediátrica de cuidados intensivos y fue sometido a hemoperfusión con carbón activado (tres horas después de su ingreso) y, luego, a hemodiafiltración veno-venosa continua por 72 horas. No requirió asistencia respiratoria mecánica invasiva, ni soporte inotrópico o vasopresor. Se practicó una endoscopia de vías digestivas altas que evidenció esofagitis de grado B y gastropatía generalizada no erosiva. Durante la hospitalización en la unidad pediátrica de cuidados intensivos, presentó un episodio de desaturación y dolor torácico. La tomografía computadorizada de vasos sanguíneos fue negativa para tromboembolismo pulmonar, pero mostró infiltrados alveolares en ambas bases pulmonares y derrame pleural bilateral sugestivo de neumonía aspirativa, edema pulmonar o hemorragia alveolar, sin signos de fibrosis. Se decidió iniciar tratamiento con ampicilina-sulbactam por siete días.

El paciente presentó mejoría clínica y egresó 18 días después del ingreso hospitalario.

### Caso 9

Se trata de un paciente de 9 años de sexo masculino, procedente de la zona rural de Argelia (Cauca), y sin antecedentes clínicos referidos. Ingresó a la institución 11 horas después de haber ingerido accidentalmente aproximadamente 15 ml de paraquat diluido en agua, con síntomas iniciales inmediatos de tres episodios eméticos.

Fue atendido en un centro médico de nivel I de atención, donde intentaron practicar un lavado gástrico y administrar carbón activado. Sin embargo, el menor no lo permitió y, por lo tanto, lo remitieron como urgencia vital. Al llegar al Hospital Universitario San José, presentaba tensión arterial sistólica de 92 mm Hg y diastólica de 52 mm Hg, frecuencia cardíaca de 81 latidos por minuto, frecuencia respiratoria de 21 respiraciones por minuto y saturación de oxígeno del 97 %. Fue trasladado a unidad pediátrica de cuidados intensivos, en donde se trató con metilprednisolona, ciclofosfamida, colchicina, N-acetilcisteína, vitamina E, ácido ascórbico y vitamina A. Se inició hemoperfusión con carbón activado luego de nueve horas del ingreso y, posteriormente, hemodiafiltración veno-venosa continua por 72 horas. No requirió asistencia respiratoria mecánica invasiva, ni soporte inotrópico o vasopresor y, durante su estancia en dicha unidad, evolucionó satisfactoriamente.

Fue dado de alta 10 días después de su ingreso hospitalario.

### Caso 10

Se trata de una paciente de 14 años de sexo femenino, procedente de la zona rural de Morales (Cauca), que ingresó a la institución con emesis en múltiples oportunidades, 11 horas después de haber ingerido aproximadamente 5 ml de paraquat.

Fue trasladada a un centro médico de nivel I de atención donde le practicaron lavado gástrico y le administraron carbón activado y, luego, fue remitida al Hospital Universitario San José. Al llegar, presentaba tensión arterial sistólica de 119 mm Hg y diastólica de 72 mm Hg, frecuencia cardíaca de 90 latidos por minuto, frecuencia respiratoria de 20 respiraciones por minuto y saturación de oxígeno del 96 %.

Fue trasladada a la unidad pediátrica de cuidados intensivos, donde recibió N-acetilcisteína, vitamina C, vitamina A, vitamina E, metilprednisolona, ciclofosfamida y colchicina. Transcurridas 14 horas desde su ingreso, se inició hemoperfusión con carbón activado y, luego, hemodiafiltración veno-venosa continua por 72 horas. Durante su estancia en dicha unidad, presentó signos de falla cardiaca manejados con propanolol y espironolactona. Luego, por fiebre y hallazgo de *Serratia marcescens* en los hemocultivos, recibió tratamiento con meropenem durante10 días con adecuada mejoría clínica. No requirió asistencia respiratoria mecánica invasiva, ni soporte inotrópico o vasopresor. Sin embargo, se diagnosticó fibrosis pulmonar con saturación de oxígeno del 82 % y disnea de pequeños a medianos esfuerzos, por lo que se sugirió una valoración por parte del grupo de trasplantes para tratar de evitar el desarrollo de hipertensión pulmonar. Se inició oxigenoterapia, con la mínima fracción inspirada de oxígeno, para mantener la saturación entre el 90 y el 92 %.

La paciente fue dada de alta después de 22 días de su ingreso hospitalario y fue remitida a un centro de IV nivel de atención para su manejo integral.

### Caso 11

Se trata de una paciente de 14 años de sexo femenino, procedente de Popayán (Cauca), que ingresó a la institución siete horas después haber ingerido aproximadamente 10 ml de paraquat, con síntomas iniciales de náuseas, sialorrea y dolor abdominal.

Fue llevada de inmediato a un centro médico de nivel I de atención, donde le practicaron un lavado gástrico y le administraron carbón activado, y luego, fue trasladada a un centro de nivel III. Al llegar al Hospital Universitario San José, presentaba frecuencia cardíaca de 84 latidos por minuto, frecuencia respiratoria de 17 respiraciones por minuto, saturación de oxígeno del 98 %, y tensión arterial sistólica de 110 mm Hg y diastólica de 62 mm Hg. Fue trasladada a la unidad pediátrica de cuidados intensivos para tratamiento con ivermectina, metilprednisolona, N-acetilcisteína, propanolol, ácido ascórbico, vitamina A, vitamina E y colchicina. Se practicó hemoperfusión con carbón activado a las seis horas después del ingreso y, luego, hemodiafiltración veno-venosa continua por 72 horas.

Tuvo una evolución clínica adecuada y fue remitida a la unidad de salud mental pediátrica.

### Caso 12

Se trata de una paciente de 16 años de sexo femenino, procedente de zona rural de Toribío (Cauca), que ingresó a la institución 23 horas después de haber ingerido aproximadamente 20 ml de paraquat.

Presentó un episodio de emesis y fue llevada a un centro médico de nivel I de atención, donde le practicaron lavado gástrico y le administraron carbón activado. Luego, fue remitida al Hospital Universitario San José. Al llegar, presentaba tensión arterial sistólica de 134 mm Hg y diastólica de 72 mm Hg, frecuencia cardíaca 88 de latidos por minuto, frecuencia respiratoria de 18 respiraciones por minuto y saturación de oxígeno del 96 %. Fue trasladada a la unidad pediátrica de cuidados intensivos, donde se inició tratamiento con metilprednisolona, ciclofosfamida, colchicina, N-acetilcisteína, vitamina E, ácido ascórbico y vitamina A. Se practicó hemoperfusión con carbón activado a las dos horas después de su ingreso y, luego, hemodiafiltración veno-venosa continua por 72 horas. No requirió asistencia respiratoria mecánica invasiva, ni soporte inotrópico o vasopresor. Con exámenes paraclínicos tomados al ingreso, se diagnosticó embarazo temprano, con alto riesgo de pérdida o de malformaciones congénitas por la ingestión de paraquat y el manejo con ciclofosfamida. Por priorización de la salud de la madre, su solicitud y la autorización del cabildo indígena al que pertenecía, se le practicó una interrupción voluntaria del embarazo. Dada la buena evolución clínica, se le dio egreso hospitalario.

### Caso 13

Se trata de una paciente de 17 años de sexo femenino, procedente de zona rural de Argelia (Cauca), sin antecedentes clínicos documentados, que ingresó a la institución 19 horas después de haber ingerido aproximadamente 20 ml de paraquat mezclado con carnes. Presentó múltiples episodios eméticos, malestar general, dolor abdominal generalizado (6/10 en la escala subjetiva del dolor) y fasciculaciones.

Consultó a un centro médico de nivel I de atención, donde la remitieron como urgencia vital. No hay datos del manejo recibido en ese centro. Al llegar al Hospital Universitario San José, presentaba tensión arterial sistólica de 140 mm Hg y diastólica de 90 mm Hg, frecuencia cardíaca de 91 latidos por minuto, frecuencia respiratoria de 29 respiraciones por minuto y saturación de oxígeno del 97 %.

Se inició tratamiento con metilprednisolona, ciclofosfamida, N-acetilcisteína, vitamina A, vitamina C, vitamina E, ivermectina y albendazol. Fue trasladada a la unidad pediátrica de cuidados intensivos, donde fue sometida a hemoperfusión con carbón activado a las 12 horas después del ingreso y, luego, a hemodiafiltración veno-venosa continua por 72 horas. No requirió asistencia respiratoria mecánica invasiva ni soporte inotrópico o vasopresor y evolucionó adecuadamente a pesar del desarrollo de una lesión renal aguda, sin complicaciones. Fue valorada por psiquiatría para tratamiento antidepresivo y trasladada a la unidad de salud mental pediátrica.

La paciente egresó, en buenas condiciones, a los 24 días después de su ingreso hospitalario.

### Caso 14

Se trata de una paciente de 17 años de sexo femenino, procedente de Popayán (Cauca), sin antecedentes de importancia, quien ingirió de forma intencional aproximadamente 15 ml de paraquat y presentó varios episodios eméticos.

Fue trasladada al Hospital Universitario San José, desde un centro médico de nivel I de atención, donde le practicaron lavado gástrico y le administraron carbón activado. Durante la remisión, la paciente presentó múltiples episodios eméticos. Al llegar, nueve horas después de la ingestión del tóxico, presentaba tensión arterial sistólica de 120 mm Hg y diastólica de 80 mm Hg, frecuencia cardíaca de 72 latidos por minuto, frecuencia de 16 respiraciones por minuto y saturación de oxígeno del 98 %.

Se inició tratamiento con metilprednisolona, ciclofosfamida, N-acetilcisteína, propanolol, colchicina, vitamina C, vitamina A. Fue trasladada a la unidad pediátrica de cuidados intensivos. En la radiografía de tórax se evidenció posible neumonitis química y se trató con ampicilina-sulbactam durante siete días. Se practicó hemoperfusión con carbón activado a las seis horas después del ingreso y se continuó con hemodiafiltración veno-venosa continua durante 72 horas. No requirió asistencia respiratoria mecánica invasiva, ni soporte inotrópico o vasopresor. En una endoscopia de vías digestivas altas, se evidenciaron erosiones en el estómago, sin úlceras ni perforaciones. Se inició nutrición enteral por sonda nasoyeyunal, que se retiró cuando toleró la vía oral. La paciente recibió apoyo psicoterapéutico y manejo de depresión con sertralina, prescrita por un psiquiatra pediátrico.

A los 15 días de su ingreso hospitalario, fue dada de alta por una adecuada evolución clínica.

### Consideraciones éticas

El reporte de esta serie de casos contó con la aprobación del Comité de Ética de Investigación Científica del Hospital Universitario San José de Popayán (aval número 16).

## Revisión de tema

Dinis-Oliveira *et al*. hicieron una amplia revisión de la literatura de la intoxicación aguda por paraquat, en la que se describe que, una vez ingerido, su absorción ocurre principalmente en el intestino delgado. En humanos, se estima que entre el 1 y el 5 % se absorbe en un periodo de 1 a 6 horas, con un pico en plasma a las 4 horas aproximadamente ^3^. En cuanto a su distribución, las concentraciones más altas de paraquat se depositan en el riñón y en los pulmones (en neumocitos de tipo I y II, y células claras renales mediante el sistema de absorción de poliaminas) ^3^. En el riñón, el paraquat se excreta normalmente mediante filtración glomerular y secreción tubular activa ^3^. Se ha planteado que también se reabsorbe en el túbulo contorneado proximal, pero aparentemente la cantidad es mínima en humanos ^3^. En el pulmón, ocurre una acumulación selectiva del paraquat y sus efectos tóxicos, por lo que se ha propuesto como un compuesto neumotóxico, pues puede alcanzar concentraciones 6 a10 veces mayores que en plasma y permanecer en el tejido pulmonar, aunque los niveles plasmáticos desciendan ^3^.

En la literatura médica, los reportes de caso de intoxicación por paraquat se remontan a 1966 ^3^. Su diagnóstico se basa en el historial de ingestión o exposición a un agroquímico desconocido, el desarrollo de quemaduras orofaríngeas graves y toxicidad sistémica ^7^.

En Colombia, el Instituto Nacional de Salud reporta la situación epidemiológica nacional de intoxicación por sustancias ^12^. Según el último boletín epidemiológico publicado del 2020 ^13^, en el 2019, la incidencia de intoxicaciones por sustancias fue de 45,1 por cada 100.000 habitantes, con el mayor número de casos en los departamentos de Quindío y Risaralda y en la ciudad de Cartagena. En este reporte, las intoxicaciones se categorizaron según sustancias psicoactivas, medicamentos, plaguicidas (categoría que incluyó al paraquat) y otras sustancias químicas ^13^. En otro informe del Instituto Nacional de Salud, gran parte de las intoxicaciones agudas por químicos en niños, desde los 10 años en adelante, fue por plaguicidas ^14^. Entre el 2018 y el 2020, las intoxicaciones por plaguicidas correspondieron al 20 a 23 % de las intoxicaciones agudas por sustancias químicas ^15^. Según la Línea Nacional de Toxicología del Ministerio de Salud y Protección Social, en el 2015, de 2.947 casos de intoxicación por plaguicidas, el 3,6 % fue por paraquat ^5^. El protocolo de vigilancia del Instituto Nacional de Salud del 2010 ^16^ y del 2014 ^17^, contempla la intoxicación de paraquat en una categoría toxicológica II y recomienda su manejo con métodos de depuración extrarrenal, como la hemoperfusión y la diálisis ^16^, en particular, en casos de ingestión masiva y durante las primeras 12 horas después de la ingestión ^5^.

Su mecanismo de toxicidad se basa en su ciclo de óxido-reducción y la subsecuente producción de especies reactivas de oxígeno (*reactive oxigen species*, ROS) y la depleción de NADPH (*nicotinamide adenine dinucleotide phosphate*) producida en la vía de la hexosa monofosfato ^3^, lo cual causa daño y muerte celular. El paraquat^2+^ es reducido por la NADPH citocromo P450 reductasa, la NADH ubicuinona oxido-reductasa, la xantina oxidasa y la óxido nítrico sintasa, para formar paraquat^+^ más NADP^+^ o NAD^+^^3^. Posteriormente, el paraquat^+^ es rápidamente reoxidado en presencia de O_2_, volviendo a su forma original (paraquat^2+^) con la subsiguiente generación de O_2_ como ROS ^3^. Otras ROS que se pueden formar a partir del O_2_^-^son el peróxido de hidrógeno (H_2_O_2_) por acción de la superóxido dismutasa, HO^-^ mediante la reacción de Haber-Weiss y de Fenton, y ONOOO^-^mediante su reacción con NO^- (^^3^. La toxicidad del paraquat depende del O_2_, por lo que el pulmón es el órgano afectado con mayor frecuencia, dada la acumulación selectiva del paraquat y la gran disponibilidad de O_2_ para la óxidoreducción cíclica ^3^.

El paraquat produce toxicidad renal directa (tubulopatía pura de predominio proximal), la cual ocurre principalmente con la ingestión de grandes cantidades, lo cual produce necrosis tubular aguda, reducción rápida del filtrado glomerular y secreción tubular y, por lo tanto, aumento de la vida media del paraquat, lo que genera un mayor efecto tóxico ^3^. La ingestión de grandes dosis de paraquat (20 mg/kg de peso) reduce la depuración renal del paraquat por la inducción de necrosis tubular aguda, en la que el gasto urinario se reduce hasta 10 a 20 veces en las primeras horas ^3^. Aunque la función renal se recupera usualmente sin secuelas, el desarrollo de lesión renal aguda impide la excreción del paraquat por la principal ruta y esto contribuye significativamente a la mortalidad ^3^. Sin embargo, aún sin lesión renal aguda, su depuración renal parece ser más lenta en humanos ^3^. Se ha planteado que, si la función renal permanece normal, aproximadamente el 90 % del paraquat es excretado de forma habitual dentro de las 12 y 24 horas después de su ingestión ^3^.

Diversos estudios en ratas con ingestión de soluciones de paraquat al 33 % o de dicloruro de paraquat al 48,6 %, demuestran que la dosis letal 50 (DL_50_) es de 276 a 344 mg/kg, por lo cual se clasifica en la categoría II de toxicidad ^2^^,^^4^. En otros reportes, la DL_50_fue de 57 a 150 mg/kg ^3^. Sin embargo, investigaciones en ratas con inhalación aguda de paraquat cristalino (DL50= 1 μg) y en conejos con exposición ocular a una solución de paraquat ionizado al 34,4 %, lo han clasificado en la categoría I de toxicidad ^2^^,^^4^ . Los reportes de monos Rhesus con exposición oral incidental a corto plazo (1 a 30 días), han demostrado un nivel mínimo de 1,5 mg de paraquat iónico/kg/día necesario para generar un efecto adverso observable ^4^. Otros estudios han informado una DL_50_ posterior a la ingestión de paraquat de 120 mg/kg en ratones, de 50 mg/kg en conejos, de 35 mg/kg en gatos, de 50 a 70 mg/kg en monos *Cynomolgus* y de 22 a 30 mg/kg en cuyes ^3^. En investigaciones en ratas, se estimó un nivel mínimo de 5 mg/kg o 3,6 mg del ión/kg para obtener un efecto adverso observable ^2^. En estos estudios, se demuestra que la DL_50_ y la dosis para un efecto adverso observable son pequeñas en las distintas especies evaluadas. Es importante aclarar que la cantidad letal ingerida para los humanos varía según el tipo de paraquat y su concentración (presentación comercial disponible). En Colombia, la presentación del 20 % para uso agrícola es la más frecuentemente utilizada y tiene por nombre comercial Gramoxone^®^, Gramafin^®^ y Gramuron^® (^^5^.

En sujetos con intoxicación aguda por ingestión, se han descrito las siguientes fases de toxicidad: intoxicación leve o asintomática con dosis ingerida inferior a 20 mg/kg de peso corporal (equivalente a menos de 7,5 ml de una solución al 20 % para un persona de 70 kg), con síntomas como náuseas, emesis, diarrea, hemorragia intestinal, hemoptisis y oliguria; intoxicación moderada a grave con dosis ingeridas de 20 a 40 mg/kg y de 40 a 50 mg/kg de peso corporal (equivalente esta última a 7,5 a 15 ml o un solo trago de una solución de 20 % para una persona de 70 kg), con síntomas como emesis, diarrea, y toxicidad sistémica, lesión renal aguda, falla hepática, hipotensión, taquicardia y muerte por falla respiratoria a causa de fibrosis pulmonar; e intoxicación fulminante con dosis ingerida superior a 40-50 mg/ kg de peso corporal (más de 15 ml de una solución al 20 % para una persona de 70 kg), con síntomas como náuseas, emesis, diarrea, falla multiorgánica y muerte antes del desarrollo de cambios radiológicos significativos ^3^^,^^5^.

Es probable la recuperación completa en el primer grupo, mientras que, en el segundo grupo, del 30 a 60 % de los pacientes mueren después del quinto día de exposición, aunque pueden sobrevivir entre 2 y 4 semanas. En el último grupo, la muerte ocurre usualmente dentro de las primeras 24 horas de la ingestión o a los pocos días (100 % entre 1 y 5 días) ^3^^,^^5^. Es más fácil que se produzca una intoxicación aguda grave o letal en pacientes pediátricos.

En la intoxicación moderada a grave, se produce irritación y hasta quemaduras en las vías digestivas altas, necrosis tubular aguda (12-48 horas después de la ingestión), hemorragia pulmonar (24-48 horas después de la ingestión) y fibrosis pulmonar (1-2 semanas después de la ingestión) ^3^.

Este grado de intoxicación comprende las siguientes tres fases. La primera se caracteriza por la presencia de lesiones corrosivas en las mucosas de las vías digestivas altas acompañadas de dolor, inflamación de la lengua (“lengua de paraquat”) y, en algunos casos, afonía y afagia ^3^. Los pacientes también pueden presentar náuseas, vómito, dolor abdominal y diarrea ^3^. En la segunda fase, entre 2 y 5 días después de la ingestión, hay desarrollo de lesión renal aguda y necrosis hepatocelular centrolobulillar con colestasis (usualmente moderada). La hipovolemia por las pérdidas gastrointestinales de fluidos y la reducción o la falta de ingestión de líquidos contribuyen al desarrollo de la lesión renal aguda ^3^. En la tercera fase, el desarrollo tardío de fibrosis pulmonar generalizada es responsable de un mal pronóstico. En su forma típica, la fibrosis se caracteriza por lesiones intersticiales que se extienden ^3^; puede llevar al rápido desarrollo de hipoxemia persistente y causar la muerte en un periodo de cinco días a muchas semanas ^3^.

En general, por los hallazgos en tomografías computadorizas pulmonares de alta resolución, se ha propuesto el desarrollo inicial de opacidades con patrón de vidrio esmerilado, seguido por consolidaciones y fibrosis pulmonar ^3^. Sin embargo, aunque la mayoría de los pacientes con cambios radiológicos pulmonares desarrollan daño pulmonar progresivo y posteriormente fatal, estos pueden ser funcional y radiológicamente reversibles ^3^.

En la intoxicación aguda por paraquat, la medición de su concentración en plasma es el método más fiable para evaluar el pronóstico como guía para el tratamiento ^3^. Se ha sugerido que los pacientes intoxicados deben ser vigilados y tratados de manera expectante, hasta que los niveles de paraquat sean reportados como inexistentes ^3^. La medición cuantitativa del paraquat mediante espectrofotometría es la técnica recomendada ^3^. Se han propuesto nomogramas con sensibilidad y especificidad alrededor del 90 %, para predecir la gravedad y la mortalidad, con base en los niveles plasmáticos de paraquat y el tiempo transcurrido desde su ingestión ^3^^,^^7^. Sin embargo, la estimación del tiempo desde la ingestión tiende a ser errada, sobre todo durante las primeras horas, cuando las concentraciones plasmáticas del paraquat declinan rápidamente. Un pequeño cambio puede alterar radicalmente la relación entre la concentración en plasma del paraquat y la línea predictiva ^3^^,^^7^. Además de otras consideraciones, como la técnica empleada para la medición del paraquat y la variación interindividual de sensibilidad ^3^, se ha encontrado que los pacientes con concentraciones plasmáticas de 10 mg/L o mayores dentro de las ocho horas después de la ingestión del paraquat, usualmente fallecen por choque cardiogénico dentro de las 24 horas posteriores a la ingestión, mientras que aquellos con concentraciones menores de 10 mg/L, pero por encima de la línea predictiva, mueren de fibrosis pulmonar y falla respiratoria posterior a las 24 horas después de la ingestión ^3^.

También, se ha propuesto el índice de gravedad de intoxicación por paraquat (*severity index of paraquat poisoning*, SIPP) basado en el tiempo en horas desde la ingestión hasta el inicio del tratamiento y las concentraciones séricas (aproximadamente tres veces menores que las concentraciones plasmáticas):

niveles séricos de paraquat 
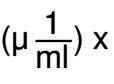
 y tratamiento (horas) ^3^.

Este índice se calculó con población adulta. Si es menor de 10, predice que los pacientes pueden sobrevivir; entre 10 y menos de 50, predice muerte tardía por falla respiratoria; y si es mayor de 50, predice una muerte temprana, secundaria a falla circulatoria ^3^. Min *et al*. reportaron un gran rendimiento del SIPP (AUC = 0,924) y de los niveles séricos de paraquat (AUC = 0,951) en población adulta, con un punto de corte mayor de 12,3 y de 2,9, respectivamente ^18^. Aunque existen otros puntajes basados en las concentraciones plasmáticas de paraquat ^3^, la medición de los niveles séricos o plasmáticos no está disponibles en todos los hospitales. Por esta razón, se ha propuesto el puntaje APACHE II en adultos, para predecir la muerte intrahospitalaria (puntaje mayor de 13, con sensibilidad del 57 % y especificidad del 94 %) ^3^.

En un estudio retrospectivo reciente de 1.199 pacientes adultos intoxicados con paraquat, Gao *et al*. propusieron un modelo predictor de mortalidad intrahospitalaria basado en ocho factores independientes de riesgo: edad, volumen de paraquat ingerido, creatina cinasa miocárdica, recuento plaquetario, recuento leucocitario, recuento de neutrófilos, gamma-glutamil transferasa y creatinina sérica ^19^. El modelo tuvo una estadística de concordancia total de 0,926 para la predicción de mortalidad intrahospitalaria en la cohorte evaluada ^19^.

Respecto al enfoque clínico y terapéutico de la población pediátrica intoxicada por paraquat, se encontró que este no difiere del de los adultos ^3^ y que requiere de atención en un centro de alta complejidad con toxicología clínica ^5^. La ventana de oportunidad para cualquier tratamiento efectivo es muy estrecha ^3^. El tratamiento exitoso de la intoxicación por paraquat consiste casi exclusivamente en medidas tempranas y agresivas de descontaminación para limitar la absorción ^3^. El paraquat se une ávidamente a la arcilla, por lo que la administración de absorbentes minerales -por vía oral o en lavados intestinales completos- puede ser útil como tratamiento prehospitalario para reducir la absorción del paraquat ^3^. Nunca debe practicarse un lavado gástrico sin la administración de un absorbente mineral ^3^. El agente absorbente de elección es el carbón activado (entre más pequeño sea el tamaño de la partícula, mejor es la remoción del tóxico por la mayor superficie de contacto), pero en caso de no estar disponible, también puede utilizarse bentonita ^3^^,^^5^. La combinación del carbón activado con citrato de magnesio puede mejorar la remoción del paraquat ^3^. Dado el pico temprano de concentración del paraquat, las intervenciones para prevenir o reducir su absorción pueden ser efectivas solo efectivas si se utilizan muy tempranamente (una a dos horas) después de la ingestión ^3^^,^^5^. Sin embargo, en la práctica clínica se administran independientemente del tiempo transcurrido entre la ingestión y el inicio del manejo. Así, cuanto hay ingestión, el manejo de la intoxicación por paraquat incluye la administración de carbón activado por vía oral o por sonda nasogástrica ^3^.

Se ha propuesto que si las concentraciones plasmáticas disminuyen por la reducción de la absorción gastrointestinal (mediante técnicas de remoción extracorpórea) durante las primeras después de la ingestión, la concentración letal no se alcanzaría en el tejido pulmonar ^3^. El único método que ha mostrado eficiencia en la eliminación extracorpórea del paraquat, es la hemoperfusión con carbón activado ^3^. Sin embargo, no se recomienda en pacientes con niveles plasmáticos de paraquat mayores o iguales a 3 mg/L, por su mal pronóstico y la ausencia de eficacia demostrada ^3^. Se ha evidenciado que un curso de 6 a 8 horas de hemoperfusión con carbón activado puede ser beneficioso si el procedimiento es aplicado dentro de las cuatro horas siguientes a la ingestión (alta concentración de paraquat en plasma) ^3^^,^^7^ e, incluso, funciona cuando las concentraciones plasmáticas son menores de 0,2 mg/L ^3^. Dada la escasa redistribución de paraquat en la circulación, se sugiere que dos o más sesiones (hasta siete sesiones) de hemoperfusión pueden ser útiles ^3^. En un estudio retrospectivo, Rao *et al*. encontraron una mayor supervivencia en aquellos pacientes que recibieron hemoperfusión con carbón activado, comparados con los que recibieron tratamiento estándar y los que iniciaron la terapia en menos de seis horas después de la ingestión del tóxico ^8^. En particular, para aquellos casos con ingestión masiva de paraquat, se recomienda practicar la hemoperfusión o la hemodiálisis en las primeras 12 horas después de la ingestión ^5^.

En un estudio retrospectivo de 207 pacientes adultos gravemente intoxicados con paraquat y manejados según el protocolo de hemoperfusión con carbón activado (dos cursos de ocho horas) y con terapia inmunosupresora, Hsu *et al*. determinaron, en el análisis ajustado que la hemoperfusión iniciada en un periodo mayor de 4 horas y menor de 5 horas reducía significativamente la mortalidad a 60 días en el subgrupo de pacientes que recibieron pulsos repetidos con inmunosupresores (metilprednisolona y ciclofosfamida, seguidos de dexametasona y repetición de la primera, de la segunda o de ambas) ^8^. En pacientes de menor edad ^3^^,^^6^^,^^19^^,^^20^, la presencia de una lesión renal aguda, una prueba cualitativa azul marino positiva para paraquat la administración de inmunosupresores en pulsos repetidos, se han visto también como variables relacionadas con una menor mortalidad ^8^.

La utilización de hemoperfusión seriada y combinada con hemodiálisis se ha recomendado durante las primeras 24 horas después de la ingestión de paraquat y cuando las concentraciones plasmáticas estén alrededor de los 10 mg/L ^3^. Sin embargo, la hemodiálisis debería ser usada en aquellos casos de lesión renal aguda inducida por paraquat ^3^. Park *et al*. identificaron que la edad, la cantidad ingerida de paraquat y la combinación concurrente de hemoperfusión y hemodiálisis, son variables independientes de mortalidad en la intoxicación por este tóxico ^21^.

En un estudio multicéntrico retrospectivo, Li *et al*. hallaron que la edad, la dosis de paraquat, los niveles séricos de paraquat al ingreso y el puntaje SOFA (*Sequential Organ Failure Assessment*) son factores de riesgo independientes, asociados con la mortalidad a los 60 días. Asimismo, la hemoperfusión, la hemofiltración veno-venosa continua o ambas, se establecieron como variables protectoras en comparación con el manejo estándar ^22^.

En un metaanálisis con tres pruebas clínicas aleatorizadas para un total de 290 pacientes incluidos con intoxicación con paraquat, Lin *et al*. no hallaron diferencias significativas entre la mortalidad del grupo control (hemoperfusión) y la de aquellos que recibieron hemoperfusión más hemofiltración venovenosa continua ^10^. Se encontró un tiempo significativamente mayor de supervivencia en quienes recibieron la intervención, pero mayor muerte por falla respiratoria ^10^. En cambio, en el grupo control, la muerte por falla circulatoria fue mayor ^10^. En un estudio clínico en el que se compararon el tratamiento estándar, la intervención con hemoperfusión y la hemofiltración venovenosa continua, Li *et al*. encontraron que la intervención es un factor protector para la mortalidad a los 90 días, la hipoxia y el desarrollo de lesión renal aguda ^23^.

En un estudio retrospectivo de 621 pacientes adultos intoxicados con paraquat, Wang *et al*. determinaron como factores independientes de supervivencia, la aplicación de hemoperfusión en menos de cuatro horas desde la ingestión de paraquat, con un segundo tiempo en menos de 20 horas, los niveles semicuantitativos de paraquat en “++” y “+++”, la aparición de lesiones pulmonares en menos de ocho días y una lesión renal aguda en etapa III ^24^.

La diuresis forzada no es muy efectiva, ya que la reabsorción tubular de paraquat es muy baja ^3^. La administración de furosemida y el reemplazo de fluidos para mantener un adecuado gasto urinario son necesarios para promover la depuración del paraquat, antes de que ocurra la lesión renal aguda ^3^.

Se han propuesto distintos fármacos para neutralizar las ROS generadas durante la intoxicación por paraquat ^3^. Sin embargo, la enzima superóxido dismutasa liposómica, los análogos solubles en agua del α-tocoferol (vitamina E) y de la vitamina E liposómica, el clofibrato, los antioxidantes de tipo tiol de bajo peso molecular, la N-acetilcisteína, los inhibidores de la xantina oxidasa (selenio, niacina, riboflavina) y los inhibidores de la enzima convertidora de la angiotensina, carecen de evidencia clínica sobre su eficacia en humanos ^3^. La administración de vitamina C no ha demostrado resultados satisfactorios, pero, con la deferoxamina, ha sido beneficiosa en modelos animales, sin pruebas aún en humanos.

Los fármacos antiinflamatorios e inmunosupresores como los esteroides (metilprednisolona) y la ciclofosfamida, tienen evidencia clínica promisoria en la prevención de la inflamación y la fibrosis pulmonar, y en la reducción de la mortalidad en la intoxicación por paraquat ^3^^,^^8^. En un estudio retrospectivo de cohorte con 1.811 pacientes adultos intoxicados por paraquat, Wu *et al*. encontraron, en el análisis ajustado, una mayor tasa de supervivencia para todos los subgrupos con tratamiento inmunosupresor que, además, recibieron metilprednisolona, ciclofosfamida o dexametasona, excepto para el subgrupo que solo recibió ciclofosfamida ^6^. La mayor supervivencia se reportó en los subgrupos de metilprednisolona más dexametasona; metilprednisolona, ciclofosfamida y dexametasona; y ciclofosfamida más dexametasona ^6^. En un metaanálisis de Cochrane con cuatro estudios clínicos aleatorizados, se estimó un bajo nivel de certeza para la reducción de mortalidad intrahospitalaria (al momento del egreso hospitalario) y a los tres meses del alta (poca diferencia en mortalidad o ninguna) en aquellos pacientes que recibieron glucocorticoides y ciclofosfamida, con respecto al grupo control (cuidado estándar) ^11^^,^^25^.

## Discusión

En los casos aquí reportados, la ingestión aproximada de paraquat varió entre 5 y 20 ml y los síntomas más frecuentes posteriores a la ingestión fueron emesis y dolor abdominal. En los tres casos en los que fallecieron los pacientes, se observó desarrollo de falla multiorgánica a los 4 o 5 días desde su ingreso a la institución, con una ingestión de 20 ml de paraquat (intoxicación fulminante en adultos) en dos de los tres casos. En 6 de los 10 casos sobrevivientes, la ingestión fue de 10 a 20 ml, y uno de los casos desarrolló fibrosis pulmonar con necesidad permanente de oxígeno suplementario y remisión a un centro de complejidad mayor para valoración de trasplante pulmonar. Sin embargo, en los demás casos no se pudo descartar el desarrollo de fibrosis pulmonar o la supervivencia sin otras complicaciones asociadas con la intoxicación, que pudieran darse después del egreso hospitalario.

Doce de los catorce pacientes eran procedentes del departamento del Cauca y el Hospital Universitario San José es un centro de referencia para la remisión de pacientes con necesidad de atención médica de mayor complejidad en esta zona del país. El protocolo institucional propuesto considera e incluye las recomendaciones hechas por el Instituto Nacional de Salud en el abordaje de las intoxicaciones agudas por paraquat ^1^^,^^5^.

La medición de las concentraciones séricas o plasmáticas del paraquat no se hizo en ninguno de los casos presentados, porque la institución no dispone de este recurso. Sin embargo, es importante considerar su medición para establecer el pronóstico del paciente al ingreso y precisar la ventana desde la ingestión del tóxico.

El puntaje propuesto por Gao *et al*. ^19^ incluye algunos exámenes paraclínicos rutinarios durante la atención de pacientes con intoxicación por paraquat. En esta serie de casos, el puntaje calculado fue de 9/14, que indica un riesgo intermedio para mortalidad intrahospitalaria. En aquellos casos de pacientes fallecidos, el puntaje tuvo un rango más amplio, con una mediana de 15 puntos (cuadro 2). Sin embargo, 15 es el puntaje límite para un volumen ingerido de paraquat de 100 ml y una edad de 50 años. Esta discrepancia se debe a que el cálculo del puntaje en los 14 casos fue incompleto, ya que en la institución no estaban disponibles las mediciones de las enzimas gammaglutamil transferasa y creatina cinasa miocárdica. Por lo tanto, el puntaje calculado en este trabajo requiere ajuste y validación en la población pediátrica para su aplicación. Según lo mencionado, no se pudo establecer un pronóstico en los casos expuestos, ni sugerir relaciones con los supervivientes o fallecidos. No se establecieron grupos con o sin terapia de remoción extracorpórea para comparación, ya que este estudio solo pretendía describir un grupo de pacientes pediátricos tratados según un algoritmo institucional de manejo para intoxicación aguda por paraquat.

**Cuadro 2:** Exámenes paraclínicos y puntaje de riesgo de mortalidad intrahospitalaria para los casos pediátricos de intoxicación aguda por paraquat (N = 14)

Variable	
Tiempo, en minutos, entre el ingreso y la toma de exámenes paraclínicos	Mediana: 40; RIC: 20-48,75
Leucocitos (1 x 10^3^ células/μl)	Media: 14,50 ± 6-32
BUN (mg/dl)	Media: 14,64 ± 7-62
Neutrófilos (1 x 10^3^ células/μΙ)	Mediana: 11,35; RIC: 5,73-18,12
Plaquetas (1 x 10^3^ células/μl)	Mediana: 294,50; RIC: 252,00- 361,00
Creatinina sérica (mg/dl)	Mediana: 0,72; RIC: 0,56-1,17
CK-MB (UI/L)	-----
GGT (UI/L)	-----
Puntaje de Gao *et al*.	Mediana: 15; RIC: 0,00-29,25

RIC: rango intercuartílico; BUN: blood urea nitrogen; CK-MB: creatine kinase-myocardial band; GGT: gamma-glutamyl transpeptidase

En 11 de los 14 casos, los pacientes se sometieron a lavado gástrico, administración de carbón activado o protección gástrica, antes de su ingreso al Hospital Universitario San José. Aunque el inicio de la terapia de remoción extracorpórea fue tardío (≥ 6 horas) en 9/12 casos, solo tres pacientes fallecieron luego de recibir el manejo basado en el protocolo institucional propuesto, en de los cuales se cumplió el protocolo completo. Por lo tanto, aún con inicio tardío de la hemoperfusión con carbón activado, la aplicación del protocolo parece ser útil en la intoxicación aguda por paraquat en población pediátrica.

En el protocolo propuesto para la población pediátrica, se encuentra el tratamiento obligatorio inmunosupresor y antioxidante junto con la eliminación sistémica del paraquat con técnicas de remoción extracorpórea ^5^^,^^6^^,^^10^^,^^11^^,^^24^. Considerando la evidencia clínica disponible en la literatura científica y la experiencia médica institucional como centro para la atención de estos casos, estos tratamientos constituyen una oportunidad de supervivencia y recuperación para el grupo de pacientes evaluado. Dos de los casos no recibieron hemoperfusión con carbón activado y uno no recibió hemodiafiltración venovenosa continua por encontrarse por fuera del periodo indicado para la remoción extracorpórea.

Como se mencionó, no se realizó la medición de los niveles de paraquat, pero próximamente podría implementarse mediante el método de ditionito sódico. No se cuenta con seguimiento a mediano ni a largo plazo de los pacientes luego de su egreso hospitalario. Se requieren estudios analíticos prospectivos en población pediátrica que permitan la comparación entre grupos y la evaluación de los puntajes propuestos para predecir resultados como la mortalidad intrahospitalaria y extrahospitalaria a largo plazo, así como el desarrollo de fibrosis pulmonar y la necesidad de trasplante de pulmón.

La ingestión, ya sea accidental o voluntaria de paraquat, se ha vuelto una causa frecuente de ingreso al Hospital Universitario San José, pero se ha logrado adquirir una experiencia valiosa con resultados satisfactorios en los niños y adolescentes atendidos, y una baja mortalidad dada la variación de la dosis de paraquat ingerida, el tiempo transcurrido hasta el ingreso y el tiempo de inicio del protocolo institucional.

Se resalta la ausencia de complicaciones asociadas con el tratamiento instaurado y, en la mayoría de los casos, una evolución clínica favorable. Este estudio sugiere la implementación de un protocolo institucional como un enfoque adecuado para reducir la mortalidad en pacientes pediátricos, al evitar el daño multiorgánico temprano y, posiblemente, la fibrosis pulmonar.
